# Supplementary material for: Analysis of PPARγ Signaling Activity in Psoriasis
Source: Int J Mol Sci. 2021 Aug 10;22(16):8603. doi: 10.3390/ijms22168603 (PMC8395241; doi:10.3390/ijms22168603)
Supplement: Supplementary file 1 [file ijms-22-08603-s001.zip › Supplemental materials_Analysis of PPARg signaling activity in psoriasis/Pathway models/Models images and html files/Differentiation of psoriatic T cells/6375.html]

IL12RB1


# Protein IL12RB1

|  |  |
| --- | --- |
| URN | urn:agi-llid:3594 |
| Total Entities | 0 |
| Connectivity | 418 |
| Name | IL12RB1 |
| Description | interleukin 12 receptor subunit beta 1 |
| Notes | The protein encoded by this gene is a type I transmembrane protein that belongs to the hemopoietin receptor superfamily. This protein binds to interleukine 12 (IL12) with a low affinity, and is thought to be a part of IL12 receptor complex. This protein forms a disulfide-linked oligomer, which is required for its IL12 binding activity. The coexpression of this and IL12RB2 proteins was shown to lead to the formation of high-affinity IL12 binding sites and reconstitution of IL12 dependent signaling. Mutations in this gene impair the development of interleukin-17-producing T lymphocytes and result in increased susceptibility to mycobacterial and Salmonella infections. Alternative splicing results in multiple transcript variants. [provided by RefSeq, Feb 2014] |
| Primary Cell Localization | Plasma membrane |
| Class | Receptor |

---

|  |  |
| --- | --- |
| Pathway | CD8+ T-Cell Activation |
|  | Th1-Cell Differentiation |
|  | Th17-Cell Differentiation |
|  | Th1-Cells Activation and Proliferation in Atherosclerosis |
|  | Lymphocyte Mediated Myocardial Injury in Myocarditis |
|  | Proteins Involved in Myocarditis |
|  | Atopic Dermatitis |
|  | T-Cells Differentiation Block in Psoriasis |
|  | Th17-Cell Function in Systemic Lupus Erythematosus |
|  | Th1-Cell Function in Systemic Lupus Erythematosus |
|  | T-Cell Cytotoxic Response against Melanocytes in Vitiligo |
|  | Toll-like Receptors in beta-Cell |
|  | CD4+T-Cell Response in Celiac Disease |
|  | Th1-Cell Activation in Crohn's Disease |
|  | Th17-Cell Activation in Crohn's Disease |
|  | Follicular Lymphoma |
|  | Il17 Signaling Related Neutrophilia in Asthma |
|  | Th17-Cell Differentiation in Asthma |
|  | Th17-Cell and Th1 Immune Responsein Psoriatic Arthritis |
|  | Th17-Cell Numbers Reduction in HIV |
|  | IL23A and IL17A Provoke Cancer-Associated Inflammation |
|  | IL12R -> STAT Signaling |
|  | IL23R -> STAT3/NF-kB Signaling |
|  | IL12R -> NF-kB/NFATC Signaling |
|  | IL12B Expression Targets |
|  | PPAR Psoriasis |
|  | 1\_Differentiation of psoriatic T cells |
|  | 1\_Defects in response to pathogens in the gut promotes inflammation in Crohn's disease |
|  | 1\_Antigen-presenting cells promote islet dysfunction and immune system activation in T1D |
|  | Differentiation of psoriatic T cells |

---

|  |  |
| --- | --- |
| MedScan ID | 3594 |
|  | 171333 |

---

|  |  |
| --- | --- |
| LocusLink ID | 3594 |
|  | 16161 |
|  | 171333 |

---

|  |  |
| --- | --- |
| Alias | IL-12 receptor beta component |
|  | IL12RB |
|  | interleukin-12 receptor subunit beta-1 |
|  | LOC270057 |
|  | interleukin IL-12R-beta1 |
|  | IL-12 receptor subunit beta-1 |
|  | interleukin IL-12 receptor beta |
|  | interleukin IL-12 receptor beta component |
|  | IL-12R subunit beta-1 |
|  | IL-12R[b] |
|  | Interleukin-12 receptor beta-1 chain precursor (IL-12R-beta1) (Interleukin-12 receptor beta) (IL-12 receptor beta component) |
|  | interleukin IL12rb I |
|  | Interleukin-12 receptor beta |
|  | IL-12 receptor beta |
|  | I12R |
|  | interleukin 12 receptor beta 1 subunit |
|  | IL12rb I |
|  | MGC34454 |
|  | IL12 receptor component |
|  | IL-12R(b) |
|  | interleukin IL-12R-beta I |
|  | interleukin IL12 receptor component |
|  | I12R\_HUMAN |
|  | LOC234387 |
|  | IL-12R-beta1 |
|  | interleukin IL-12R(b) |
|  | CD212 antigen |
|  | IL12rb1 |
|  | interleukin IL12rb1 |
|  | interleukin IL12 receptor, beta 1 |
|  | antigen CD212 |
|  | interleukin IL-12R[b] |
|  | interleukin 12 receptor, beta 1 |
|  | CD212 |
|  | interleukin IL12RB |
|  | cluster of differentiation 212 |
|  | IL-12R-beta I |
|  | IMD30 |
|  | interleukin-12 receptor beta-1 chain |
|  | IL-12R-beta-1 |
|  | IL-12RB1 |

---

|  |  |
| --- | --- |
| GO ID | 0019955 |
|  | 0004896 |
|  | 0016517 |
|  | 0005143 |
|  | 0042019 |
|  | 0042020 |
|  | 0071346 |
|  | 0019221 |
|  | 0035722 |
|  | 0038155 |
|  | 0001916 |
|  | 0002827 |
|  | 2000330 |
|  | 2000318 |
|  | 0042104 |
|  | 0002230 |
|  | 0032729 |
|  | 0043382 |
|  | 0007165 |
|  | 0009897 |
|  | 0042022 |
|  | 0072536 |
|  | 0005886 |
|  | 0043235 |
|  | 0018108 |
|  | 0016021 |
|  | 0016020 |
|  | 0004872 |

---

|  |  |
| --- | --- |
| KEGG ID | hsa:3594 |
|  | mmu:16161 |
|  | rno:171333 |

---

|  |  |
| --- | --- |
| Organism | Homo sapiens {Organism urn:agi-taxid:9606} |
|  | Mus musculus {Organism urn:agi-taxid:10090} |
|  | Rattus norvegicus {Organism urn:agi-taxid:10116} |
|  | Mus musculus |
|  | Homo sapiens |
|  | Rattus norvegicus |

---

|  |  |
| --- | --- |
| Mouse chromosome position | 8 34.21 cM |
|  | 8 33.5 cM |

---

|  |  |
| --- | --- |
| OMIM ID | 601604 |
|  | 614891 |
|  | 209950 |

---

|  |  |
| --- | --- |
| Rat chromosome position | 16p14 |

---

|  |  |
| --- | --- |
| Hugo ID | 5971 |
|  | HGNC:5971 |

---

|  |  |
| --- | --- |
| Human chromosome position | 19p13.11 |
|  | 19p13.1 |

---

|  |  |
| --- | --- |
| Swiss-Prot Accession | P42701 |
|  | P42701.1 |
|  | Q60837 |
|  | A0A1D5RL98 |
|  | Q60837.2 |
|  | E9PSU7 |
|  | A8K308 |
|  | B2RPF1 |
|  | B7ZKK3 |
|  | Q8N6Q7 |
|  | Q3UV59 |
|  | Q3TAD0 |
|  | Q6PG56 |

---

|  |  |
| --- | --- |
| PIR ID | I37892 |
|  | I49295 |

---

|  |  |
| --- | --- |
| GenBank ID | NC\_000019 |
|  | XM\_011527966 |
|  | XP\_011526268 |
|  | XM\_011527967 |
|  | XP\_011526269 |
|  | XM\_011527968 |
|  | XP\_011526270 |
|  | XM\_011527969 |
|  | XP\_011526271 |
|  | XM\_011527974 |
|  | XP\_011526276 |
|  | XM\_011527973 |
|  | XP\_011526275 |
|  | XM\_017026762 |
|  | XP\_016882251 |
|  | XM\_011527970 |
|  | XP\_011526272 |
|  | XM\_011527972 |
|  | XP\_011526274 |
|  | XM\_011527971 |
|  | XP\_011526273 |
|  | XM\_006722741 |
|  | XP\_006722804 |
|  | XM\_011527975 |
|  | XP\_011526277 |
|  | NM\_001290024 |
|  | NP\_001276953 |
|  | NM\_001290023 |
|  | NP\_001276952 |
|  | NM\_005535 |
|  | NP\_005526 |
|  | XM\_011527976 |
|  | XP\_011526278 |
|  | XM\_011527977 |
|  | XP\_011526279 |
|  | NM\_153701 |
|  | NP\_714912 |
|  | NG\_007366 |
|  | AC020904 |
|  | AJ297688 |
|  | CAC10446 |
|  | AJ297689 |
|  | AJ297690 |
|  | AJ297691 |
|  | AJ297692 |
|  | AJ297693 |
|  | AJ297694 |
|  | AJ297695 |
|  | AJ297696 |
|  | AJ297697 |
|  | AJ297698 |
|  | AJ297699 |
|  | AJ297700 |
|  | AJ297701 |
|  | AY771996 |
|  | AAV28734 |
|  | CH471106 |
|  | EAW84653 |
|  | EAW84654 |
|  | EAW84655 |
|  | EAW84656 |
|  | AI637915 |
|  | AK225919 |
|  | AK290423 |
|  | BAF83112 |
|  | BC029121 |
|  | AAH29121 |
|  | BC137404 |
|  | AAI37405 |
|  | BC137406 |
|  | AAI37407 |
|  | BX647221 |
|  | DA008204 |
|  | U03187 |
|  | AAA21340 |
|  | P42701 |
|  | NC\_000074 |
|  | XM\_006509559 |
|  | XP\_006509622 |
|  | XM\_006509557 |
|  | XP\_006509620 |
|  | XM\_006509560 |
|  | XP\_006509623 |
|  | NM\_008353 |
|  | NP\_032379 |
|  | XM\_006509558 |
|  | XP\_006509621 |
|  | XM\_006509561 |
|  | XP\_006509624 |
|  | XM\_011242266 |
|  | XP\_011240568 |
|  | XM\_006509562 |
|  | XP\_006509625 |
|  | AC162446 |
|  | AY170846 |
|  | CH466569 |
|  | EDL28875 |
|  | EDL28876 |
|  | AK137577 |
|  | BAE23414 |
|  | AK171937 |
|  | BAE42738 |
|  | U23922 |
|  | AAA87457 |
|  | Q60837 |
|  | NC\_005115 |
|  | XM\_006252821 |
|  | XP\_006252883 |
|  | NM\_001170604 |
|  | NP\_001164075 |
|  | AC\_000084 |
|  | AABR07024875 |
|  | AAHX01086843 |
|  | CH474031 |
|  | EDL90740 |
|  | AF083328 |
|  | AAD16039 |
|  | NC\_018930 |
|  | AMYH02035720 |
|  | AMYH02035721 |
|  | AMYH02035722 |
|  | AMYH02035723 |
|  | EU832463 |
|  | ACE87635 |
|  | EU832511 |
|  | ACE87683 |
|  | EU832545 |
|  | ACE86947 |
|  | EU832592 |
|  | ACE86994 |
|  | JF432889 |
|  | ADZ16106 |
|  | AC\_000030 |
|  | AAHY01071879 |
|  | BC057212 |
|  | AAH57212 |
|  | XM\_005259893 |
|  | XP\_005259950 |
|  | AC\_000151 |
|  | ABBA01051245 |
|  | ABBA01051246 |
|  | NT\_011295 |
|  | NW\_927195 |
|  | NW\_001838484 |
|  | AC\_000062 |
|  | NT\_039462 |
|  | NW\_001030897 |
|  | Q3TAD0 |
|  | Q3UV59 |
|  | Q6PG56 |

---

|  |  |
| --- | --- |
| Swiss-Prot ID | I12R1\_HUMAN |
|  | I12R1\_MOUSE |
|  | Q3UV59\_MOUSE |

---

|  |  |
| --- | --- |
| Cell Localization | Membrane |
|  | Plasma membrane |

---

|  |  |
| --- | --- |
| Ensembl ID | ENSG00000096996 |
|  | ENSP00000472165.2 |
|  | ENST00000593993.6 |
|  | ENSP00000314425.5 |
|  | ENST00000322153.11 |
|  | ENSMUSG00000000791 |
|  | ENSMUSP00000000808.7 |
|  | ENSMUST00000000808.7 |
|  | ENSMUSP00000148279.1 |
|  | ENSMUST00000212657.1 |
|  | ENSRNOG00000019216 |
|  | ENSRNOP00000025977.6 |
|  | ENSRNOT00000025977.6 |
|  | ENSP00000472165 |
|  | ENST00000593993 |
|  | ENSP00000314425 |
|  | ENST00000322153 |
|  | ENSMUSP00000000808 |
|  | ENSMUST00000000808 |
|  | ENSRNOP00000025977 |
|  | ENSRNOT00000025977 |

---

|  |  |
| --- | --- |
| MGI ID | MGI:104579 |
|  | 104579 |

---

|  |  |
| --- | --- |
| RGD ID | 620025 |

---

|  |  |
| --- | --- |
| Unigene ID | Hs.567294 |
|  | Mm.731 |
|  | Rn.162266 |
|  | Rn.233637 |

---

|  |  |
| --- | --- |
| Homologene ID | 4042 |

---

|  |  |
| --- | --- |
| Shape | Stick-vertex |

---

|  |  |
| --- | --- |
| IPI ID | IPI00121384 |
|  | IPI00001400 |
|  | IPI00218703 |
|  | IPI00297449 |
|  | IPI00877269 |
|  | IPI00795120 |
|  | IPI00461866 |

---
